# A Case of Black Esophagus

**DOI:** 10.7759/cureus.5577

**Published:** 2019-09-05

**Authors:** Ibrahim Haddad, Mohammad Alomari, Bara El Kurdi, Laith Al Momani, Madhusudhan R Sanaka

**Affiliations:** 1 Internal Medicine, Quillen College of Medicine, East Tennessee State University, Johnson City, USA; 2 Internal Medicine, Cleveland Clinic Foundation, Johnson City, USA; 3 Gastroenterology, University of Missouri Kansas City (UMKC), Kansas City, USA; 4 Gastroenterology and Hepatology, Cleveland Clinic Foundation, Johnson City, USA

**Keywords:** acute esophageal necrosis, black esophagus, esophagogastroduodenoscopy

## Abstract

Acute esophageal necrosis, commonly known as black esophagus, is a serious clinical condition that requires prompt diagnosis and management to improve morbidity and mortality. We present a 47-year-old woman who had this potentially lethal condition. The patient initially presented with hematemesis, and esophagogastroduodenoscopy at presentation showed diffuse esophageal ulcerations, erosions, and necrosis. During her admission, she required multiple blood transfusions for active bleeding, after which her clinical condition stabilized. Repeat esophagogastroduodenoscopy showed near-complete resolution of the earlier findings.

## Introduction

Acute esophageal necrosis is a rare clinical diagnosis that was first described during a postmortem in 1967 by Brennan [1]. Since then, only a few cases have been reported in the literature. It is hypothesized that the condition arises from a combination of ischemic mucosal injury seen in shock states conjoined with chemical injury from the gastric contents, particularly in malnourished individuals with a functional or organic gastric outlet obstruction. Clinically, affected patients present with upper gastrointestinal bleeding with variable degrees of hemodynamic instability. Endoscopic appearance of circumferential black mucosal discoloration of the distal esophagus constitutes the hallmark finding to diagnose this syndrome.

## Case presentation

**Figure 1:**
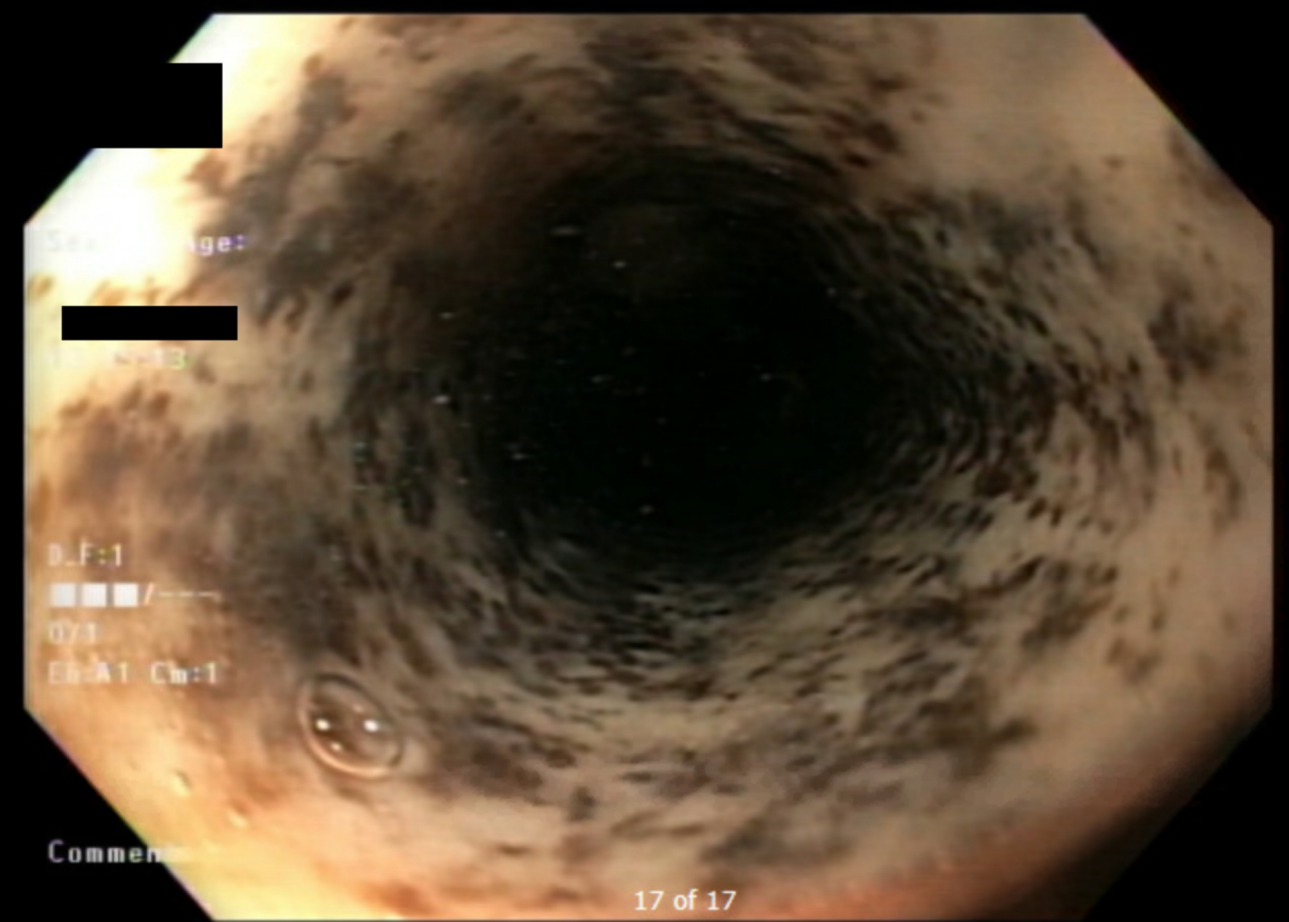
Esophagogastroduodenoscopy at presentation

**Figure 2:**
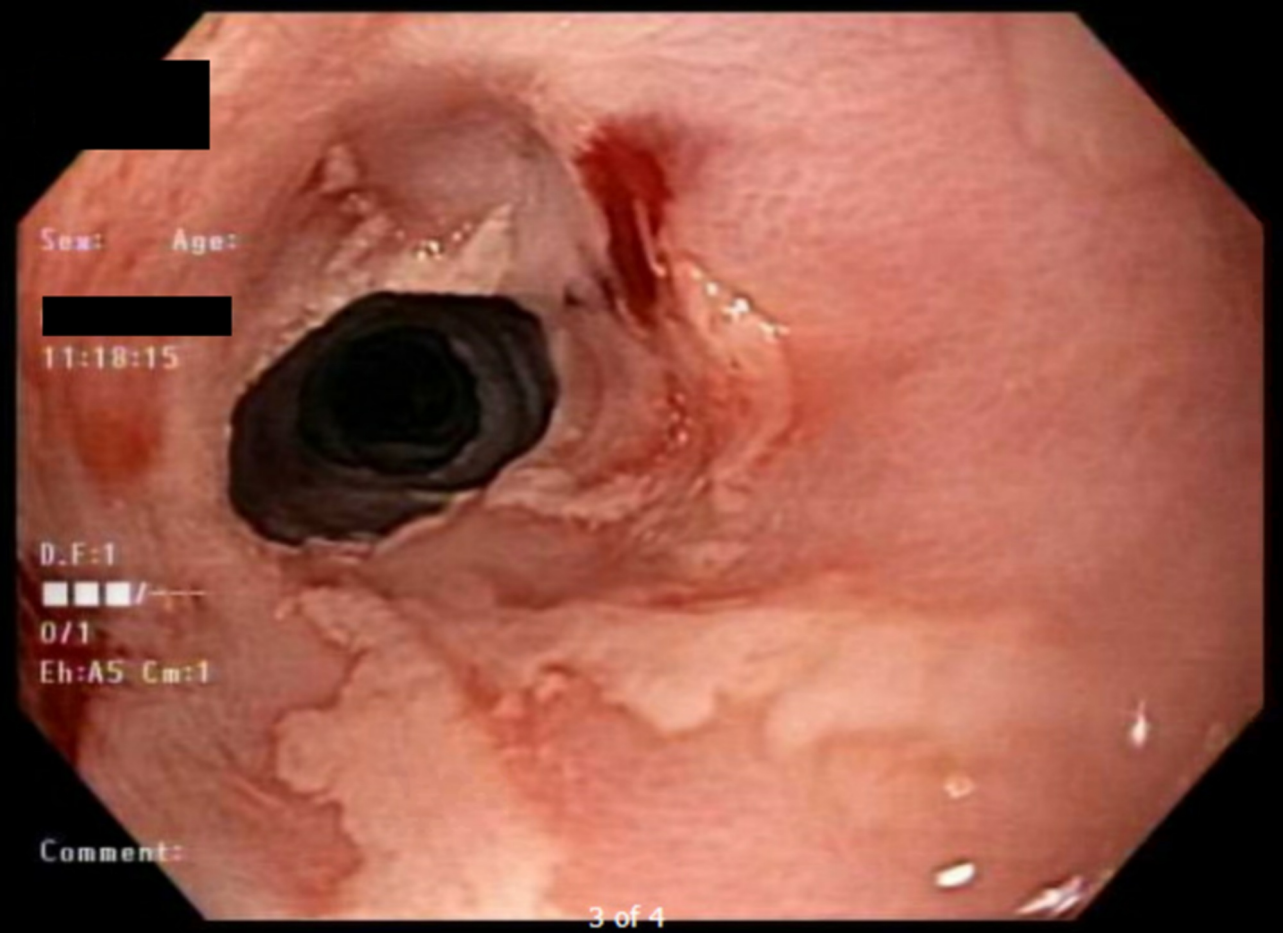
Esophagogastroduodenoscopy two weeks following discharge

A 47-year-old woman with a past medical history significant for alcohol use disorder, gastroesophageal reflux disease, and severe malnutrition presented to our institution with hematemesis following multiple episodes of induced vomiting. When asked, her rationale was to detoxify her body from recent heavy drinking. At presentation, the patient was hypotensive and required multiple normal saline boluses. She was also started on intravenous omeprazole and admitted to the intensive care unit. A complete blood count showed a drop in her hemoglobin level from a baseline of 9 mg/dl (normal range, 12 to 15.5 mg/dl) to 4.6 mg/dl; hence, she was transfused two units of packed red blood cells. After clinical stabilization, urgent esophagogastroduodenoscopy (EGD) was performed and showed diffuse esophageal ulcerations, erosions, and necrosis; these findings are consistent with acute esophageal necrosis (Figure 1) and active diffuse gastric bleeding. The patient’s hemoglobin continued to drop and fell below 4 mg/dl, necessitating a massive blood transfusion. Shortly after, she was intubated for airway protection and started on vasopressors for her refractory hemorrhage. Four days later, the patient’s blood pressure normalized, with no further drop in her hemoglobin, and she was successfully extubated. She was discharged to her home on an oral proton pump inhibitor and sucralfate. Follow-up EGD performed two weeks after discharge (Figure 2) revealed a significant interval improvement in the previously noted mucosal changes.

## Discussion

Acute esophageal necrosis is a rare entity. In one retrospective study that reviewed 10,295 upper gastrointestinal endoscopies that were carried out over five years, the incidence was found to be 0.28%, with an average age of 75 years [2]. Nonetheless, many experts believe that the reported incidence underestimate the true incidence of this condition due to the transient nature and spontaneous resolution of this condition. Previous reports showed that it is more common in geriatric males, with a male-to-female ratio of 2.3:1 [3]. Although it can involve variable lengths of the esophagus, the distal part is the most commonly affected [4].

Acute esophageal necrosis is believed to be multifactorial in origin. Factors such as an ischemic insult, corrosive injury from gastric contents, and decreased function of the mucosal barrier play a significant role in its pathogenesis. Risk factors include severe sepsis, shock, diabetic ketoacidosis, acute alcoholic intoxication, alcohol abuse, gastric volvulus, post-traumatic aortic injuries, aortic dissection, thromboembolism, and neoplastic diseases. A retrospective study [5] that evaluated the clinical presentation of these patients reported hematemesis as the most common presenting symptom, with an incidence of 66% followed by shock 36%, melena 33%, and abdominal or substernal pain 28%.

Diagnosis is established via characteristic endoscopic findings. Treatment is focused on treating the underlying conditions with the necessary supportive care. It is also essential to keep those patients fasting in addition to aggressive fluid and blood transfusion to improve blood supply and to correct the hemodynamic compromise. Gastric acid suppression and mucosal protection with intravenous proton pump inhibitors, and oral sucralfate suspension are paramount to prevent further damage from gastric acid. Due to the risk of esophageal perforation, nasogastric tube insertion is not recommended [6]. Rarely, surgical management with esophagectomy has been described when complicated by a full-thickness perforation [7]. Superinfection of the necrotic material should be suspected in case of clinical deterioration or signs of infection [8].

The outcome depends mainly on the underlying cause, with a mortality rate of 30% to 50% [4]. Acute complications include bleeding, superinfection, and perforation, with perforation being the most severe complication [9]. Long-term complications include esophageal stenosis and stricture formation.

## Conclusions

Black esophagus is a rare but potentially lethal condition that is believed to be multifactorial in origin, with a mortality rate ranging from 30% to 50%. Complications may be acute such as bleeding, mediastinitis, and perforation or chronic-like stricture formation and stenosis. The mainstays of treatment are correcting the underlying conditions, improving blood supply, and acid suppression therapy.
